# Mammalian MCM Loading in Late-G_1_ Coincides with Rb Hyperphosphorylation and the Transition to Post-Transcriptional Control of Progression into S-Phase

**DOI:** 10.1371/journal.pone.0005462

**Published:** 2009-05-07

**Authors:** Piyali Mukherjee, Thinh V. Cao, Sherry L. Winter, Mark G. Alexandrow

**Affiliations:** Program in Molecular Oncology, Moffitt Cancer Center and Research Institute, University of South Florida, Tampa, Florida, United States of America; University of Minnesota, United States of America

## Abstract

**Background:**

Control of the onset of DNA synthesis in mammalian cells requires the coordinated assembly and activation of the pre-Replication Complex. In order to understand the regulatory events controlling preRC dynamics, we have investigated how the timing of preRC assembly relates temporally to other biochemical events governing progress into S-phase.

**Methodology/Principal Finding:**

In murine and Chinese hamster (CHO) cells released from quiescence, the loading of the replicative MCM helicase onto chromatin occurs in the final 3–4 hrs of G_1_. Cdc45 and PCNA, both of which are required for G_1_-S transit, bind to chromatin at the G_1_-S transition or even earlier in G_1_, when MCMs load. An RNA polymerase II inhibitor (DRB) was added to synchronized murine keratinocytes to show that they are no longer dependent on new mRNA synthesis 3–4 hrs prior to S-phase entry, which is also true for CHO and human cells. Further, CHO cells can progress into S-phase on time, and complete S-phase, under conditions where new mRNA synthesis is significantly compromised, and such mRNA suppression causes no adverse effects on preRC dynamics prior to, or during, S-phase progression. Even more intriguing, hyperphosphorylation of Rb coincides with the start of MCM loading and, paradoxically, with the time in late-G_1_ when *de novo* mRNA synthesis is no longer rate limiting for progression into S-phase.

**Conclusions/Significance:**

MCM, Cdc45, and PCNA loading, and the subsequent transit through G_1_-S, do not depend on concurrent new mRNA synthesis. These results indicate that mammalian cells pass through a distinct transition in late-G_1_ at which time Rb becomes hyperphosphorylated and MCM loading commences, but that after this transition the control of MCM, Cdc45, and PCNA loading and the onset of DNA replication are regulated at the post-transcriptional level.

## Introduction

The molecular events involved in regulating the entry of mammalian cells into the cell cycle and eventually into S-phase are controlled by soluble growth factors that initiate signals during the first gap (G_1_) phase of their division cycle. A key component of mammalian cells that regulates entry into S-phase, and whose timely assembly and activation is likely controlled by these growth factor-induced signals, is the pre-Replication Complex (preRC) [1]. The preRC marks origins of DNA replication and controls activation of bidirectional DNA replication from these origins once S-phase is initiated. The assembly of the preRC involves the stepwise recruitment of multiple proteins, the nucleation of which begins with the arrival of the Origin Recognition Complex (ORC) [2]. This is followed by recruitment of Cdt1 and Cdc6, which together facilitate the loading of the Mini-Chromosome Maintenance (MCM) complex onto chromatin at the preRC [3], [4], [5], [6], [7]. The MCM complex is involved in the unwinding of origin DNA and is required for elongation of replication forks, strongly implicating it as the replicative helicase [8], [9]. Activation of the MCM complex requires the recruitment of Cdc45, an apparent cofactor for MCM function during initiation and elongation steps [8]. PCNA and DNA polymerases are also recruited prior to initiating DNA synthesis [10]. In cycling cells, the preRC assembles during late telophase (mitosis) [11], [12], but evidence suggests that in mammalian cells released from quiescence the loading of MCMs (final preRC assembly) occurs during late-G_1_-phase [13], [14], [15], [16]. This is supported by the results of Mailand and Diffley [17] where it was shown that Cyclin E/Cdk2 activity, which is active in middle to late-G_1_ in cells released from quiescence (see below), phosphorylates Cdc6 to achieve Cdc6-dependent MCM loading.

Progress through G_1_ into S-phase is governed by cyclin proteins that regulate associated kinases, and the temporal activation of these kinases properly orchestrates important cell cycle events as cells progress into S-phase. Included among these kinase complexes are: Cyclin D/Cdk4, Cyclin E/Cdk2, and Cyclin A/Cdk2 [18]. Entry into G_1_ from a quiescent state (G_0_) is associated with the expression and activation of Cyclin D/Cdk4, which causes an initial phosphorylation of the retinoblastoma protein (Rb) during the first half of G_1_ [19], [20], [21], [22], [23], [24]. This hypophosphorylated form of Rb is now capable of binding to E2F family members, resulting in suppression of their transcriptional transactivation potential during early G_1_ [21], [25]. In late-G_1_, Cyclin E/Cdk2 complexes form and further phosphorylate Rb (in addition to their role in Cdc6 phosphorylation and MCM loading), which produces a hyperphosphorylated form of Rb that is inactivated with respect to its ability to suppress E2F function [20], [24]. Such E2F complexes that are no longer suppressed by Rb become transactivators at the transcriptional level of genes whose protein products are required for entry into S-phase [26]. Although there are likely other non-transcriptional functions of Rb that are altered by its hyperphosphorylation [27], it is generally thought that the transcriptional activation of new mRNA in late-G_1_ for E2F-regulated genes is critical in promoting the final progression into S-phase. As a corollary, such transcription by E2F complexes is predicted to be required for preRC assembly in late-G_1_.

It has been known for almost three decades that mouse fibroblasts lose the requirement for ongoing *de novo* synthesis of mRNA in late-G_1_, approximately 3–4 hours prior to S-phase entry [28], [29], [30]. At such time, previous studies have shown that mammalian cells become insensitive to inhibitors of RNA polymerase II, such as α-amanitin or 5,6-dichloro-ribofuranosyl-benzimidazole (DRB) [28], [29], [30]. These results indicate that mammalian cells have generated the minimum amount of coding mRNA necessary for G_1_-S transit *prior* to 3–4 hours before S-phase entry, and no longer need any new mRNA production in late-G_1_.

Intriguingly, these prior results predict that the loss of need for *de novo* mRNA synthesis in late-G_1_ may overlap the window when Rb becomes hyperphosphorylated and transcriptional induction of various E2F-regulated mRNAs would be turned on and presumably required. As such, this creates a potential conflict for the Rb-E2F transcriptional induction paradigm, where ongoing late-G_1_ *de novo* transcription is thought to be required for G_1_-S transit (and consequently also for preRC assembly). Further, given that MCM assembly likely occurs in late-G_1_ after Cyclin E/Cdk2-dependent Cdc6 phosphorylation [17], another prediction that can be made from prior studies is that this late-G_1_ independence from *de novo* mRNA synthesis also potentially overlaps the time when preRCs assemble. This would indicate that ongoing new mRNA synthesis (including by E2F) is not required for preRC assembly, consistent with it not being required for G_1_-S transit. However, at the moment, any overlaps of such events are only predictions that can be made from separate reports in the literature, and have not been directly investigated together experimentally. Clearly, elucidation of the dynamics and kinetics of these events during G_1_-to-S progression will undoubtedly have important implications for understanding cell cycle control.

To address these predicted potential overlaps in a comprehensive manner with direct experimentation, we have utilized two model mammalian cell lines to investigate the relationship between preRC assembly dynamics, Rb hypo- and hyperphosphorylation, and the window of time during which cells become insensitive to the suppression of new mRNA synthesis. Using effective synchrony regimens, we have found that in mammalian cells released from quiescence the loading of MCM proteins onto pre-established ORCs begins at 3–4 hours prior to G_1_-S, consistent with the timing predicted by the Mailand and Diffley report [17]. When MCM chromatin loading is first observed, several events do indeed coincide. Rb becomes noticeably hyperphosphorylated, and, paradoxically, mammalian cells *then* lose the requirement for ongoing *de novo* synthesis of mRNA (including that of multiple E2F-regulated targets that were analyzed). We further show that mammalian cells not only transit into S-phase under conditions of significantly suppressed mRNA synthesis, but also enter on time and progress through the majority of S-phase unhindered. Suppression of mRNA synthesis from late-G_1_ onward does not perturb any measured aspects of preRC dynamics, including the loading and maintenance of MCMs, Cdc45, and PCNA on chromatin. Consistent with numerous predictions from the literature, these results provide direct experimental evidence demonstrating that mammalian cells pass through a unique transition in the cell cycle that occurs several hours prior to S-phase and coincides with Rb hyperphosphorylation and the start of MCM loading. From this transition forward, new mRNA synthesis is not rate limiting for MCM, Cdc45, or PCNA loading, nor for the onset of DNA replication.

## Methods

### Cell Culture and Synchronization

Mouse keratinocytes (Balb/MK) were maintained in low calcium MEM and supplemented with 8% dialyzed FCS (Hyclone) and 4 ng/ml EGF (Invitrogen) [30], [31]. Chinese hamster ovary (CHO) cells were maintained in normal MEM supplemented with 10% Fetal Clone II (Hyclone) [14]. MCF7 cells were maintained in DMEM (Invitrogen) and 10% FBS (Hyclone). All cells were cultured in a humidified 5% CO_2_ environment. Synchronization of CHO cells in a quiescent state was achieved by culturing cells for 36 hours in isoleucine-minus MEM supplemented with 10% dialyzed FCS [14]. Synchronization of Balb/MK cells in G_0_ was achieved by culturing cells in medium lacking EGF for 3.5 days [30], [31]. Cells were re-stimulated to enter the cell cycle (into G_1_) by addition of isoleucine-containing MEM or medium containing EGF.

### Nuclear Labeling and Flow Cytometric Analyses

Replicating DNA was labeled by either pulsing for 30 minutes with bromodeoxyuridine (BrdU; 15 µM) at the indicated time points, or by continuous labeling with BrdU (20 µM) throughout the experiment. For time point collection, labeled nuclei were fixed with 2% formaldehyde at the conclusion of the pulse with BrdU and stored until all time points were collected. Incorporated BrdU was detected using immunofluorescent approaches [14], [15]. For flow cytometry, cells were trypsinized, collected, and fixed in cold 70% ethanol at each time point. Fixed cells were stained with propidium iodide and treated with RNAse A (Sigma) prior to analysis.

### Uridine Incorporation Assays

CHO cells were pulsed with 3 µCi/ml of tritiated-uridine for 1 hr. Pulses were stopped by addition of 1 M citric acid to the medium. Following three washes with 10% trichloroacetic acid, labeled cells were lysed with 0.2 N NaOH and equal aliquots were measured by scintillation counting of duplicate samples.

### In Vivo RNA Run-off Assays

A published protocol was used with some modifications [32]. After DRB or DMSO treatment of CHO cells, bromo-uridine (BrU; Sigma) was added at 100 µM for 2 hr to label newly-synthesizing RNA. Total RNA was purified as described [33]. RNA was heated to 80 C to denature and subjected to immunopurification in 1×PBS for 1 hr at room temperature with anti-BrdU antibodies (1 µg/750 µl final; Roche) in the presence of ∼10 µg of HeLa total RNA per reaction, and then 1 hr with anti-mouse secondary agarose beads (Sigma) pre-blocked with HeLa total RNA and 0.1% BSA. Flow-through was kept, and beads were washed three times, followed by boiling in DEPC-water. RT-PCR was performed as described below.

### Antibodies Used

The following antibodies were used, with dilutions indicated. Developed by us using full-length immunogens: rabbit anti-Mcm2 (CHO samples only; 1∶5,000; Covance Labs) and chicken anti-Cdc45 (1∶1000; Aves Labs); from Cell Signaling: rabbit anti-Rb-P-ser807/811 and rabbit anti-Rb-P-ser780 (both 1∶500); from Calbiochem: monoclonal anti-PCNA (1∶10,000); from Upstate: rabbit anti-Cyclin E and rabbit anti-Cyclin A (both 1∶1000; CHO samples only); from Santa Cruz Biotech: monoclonal anti-Lamin A/C (1∶200); from BD Biosciences: monoclonal anti-Orc4 (1∶1000), rabbit anti-Mcm2 (MK samples only; 1∶3000), and monoclonal anti-Cyclin E (MK samples only; 1∶1000). From Neomarkers (Thermo-Fisher): monoclonal anti-Cyclin A (MK samples only; 1∶1000); provided by Rolf Knippers (Konstanz, Germany): rabbit anti-Orc2 (1∶1000) and rabbit anti-Mcm5 (1∶3000); provided by Steve Hann (Vanderbilt University): rabbit anti-Myc (1∶500).

### Reverse Transcriptase PCR

Total RNA was collected by standard techniques [33] and converted to cDNA. PCR was performed using Taq polymerase (Promega) and internal primers against the c-*myc*, Cyclin A2, Cyclin E1, Cdc6, E2F1, DHFR, and PCNA coding sequences. Primers were designed against Chinese hamster coding sequences (for DHFR, Cdc6, and PCNA), or against conserved regions of human and mouse coding sequences (for Cyclins A2 and E1, c-*myc*, and E2F1). PCR was performed in triplicate using multiple amplification cycle numbers (*e.g.*, 25, 27, 30 cycles), and in all cases shown, the results were obtained from the lowest number of cycles and are below saturation kinetics. Further PCR conditions and primer sequences are available upon request.

### Immunoblotting Assays

Synchronous cells were washed and scraped into cold PBS. To determine the total number of cells collected, an aliquot of scraped cells was removed and resuspended in a HEPES-buffered solution (pH 7.5) containing 10 mM EDTA to disaggregate cells (15 min on ice) [14], [15]. The approximate cell numbers collected were determined using a hemacytometer, and samples were then normalized to cell number (cell numbers never varied by more than 5%). Equal cell numbers were lysed and boiled directly in loading dye (for total lysates; TCE samples), or were separated into detergent-resistant (referred to as P3) or detergent-soluble (referred to as S1) fractions as described previously [12], [14], [15]. The detergent-resistant pellets are operationally defined as chromatin-bound, while the detergent-soluble fraction contains nucleosolic and cytosolic proteins. Subunits of the preRC that are present in the P3/chromatin fraction have been shown to be sensitive to nuclease digestion and are extractable following such a procedure [12]. Thus, the S1/P3 pairs of samples had equal volumes of CHO or Balb/MK cell-equivalent extracts, which were also equivalent to the TCE lysates. Equal amounts of TCE, S1, and P3 were analyzed by SDS-PAGE and immunoblotting. Standard immunoblotting techniques were used [34].

## Results

### Balb/mouse keratinocytes cells lose the requirement for *de novo* mRNA synthesis 4 hrs prior to S-phase

It has been known for almost three decades that mouse fibroblasts (AKR-2B, A31, and BPA-31; latter two related to 3T3 cells) released from quiescence (G_0_) enter into a unique biochemical state in late-G_1_ in which they are no longer dependent on *de novo* synthesis of coding mRNA [28], [29], [30]. This was shown by determining the times in G_1_ when cells become insensitive to two potent and specific inhibitors of RNA polymerase II function: α-amanitin or 5,6-dichloro-ribofuranosyl-benzimidazole (DRB) [35], [36]. Cells are highly sensitive to DRB-mediated mRNA suppression in early-G_1_, but become DRB-insensitive approximately 3–4 hours prior to the time of S-phase entry [28], [29], [30]. Thus, ongoing new mRNA synthesis is absolutely required in early-G_1_ and is rate-limiting for cell cycle progression during this time, but new mRNA synthesis is not rate-limiting in late-G_1_ for cell cycle progression (into S-phase).

**Figure 1:**
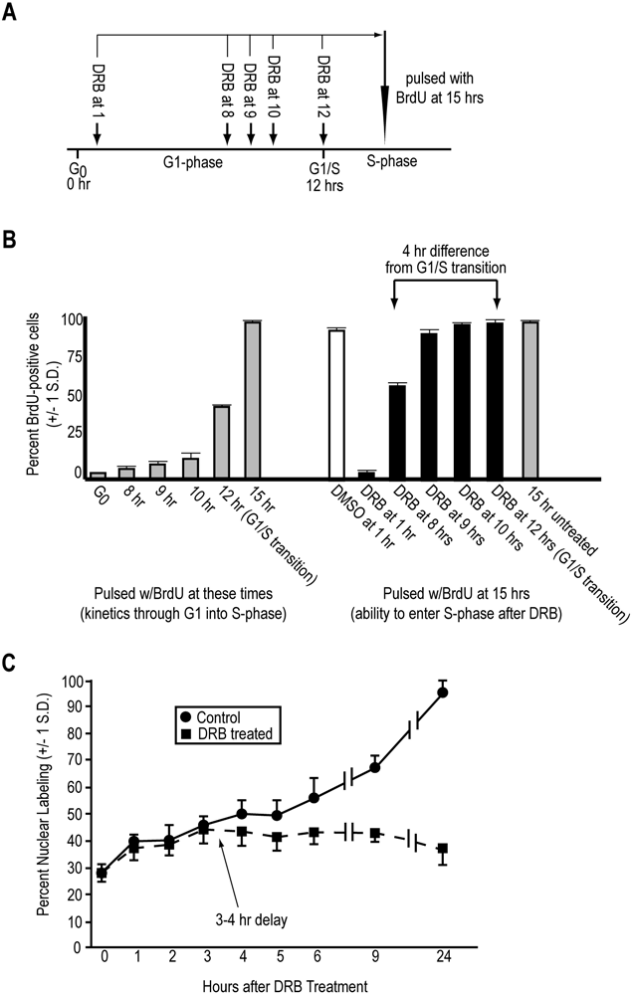
*De novo* mRNA synthesis is not required in the final 3–4 hrs of G_1_ for entry into S-phase. (*A*) Diagram illustrating the experimental design for the data obtained in *B*. (*B*) Balb/MK cells were synchronized in G_0_ by EGF deprivation and then released into the cell cycle by re-addition of EGF. At the times indicated, control cells were pulsed with BrdU to determine the kinetics of progression through G_1_ into S-phase (gray columns on left). Cells treated with 50 µM DRB at the times indicated were allowed to progress to the peak of S-phase at 15 hrs, at which time they were pulsed with BrdU to determine the percentage of cells that could enter S-phase following different times of DRB exposure (black columns on right). As a control, the DMSO carrier was added to a parallel culture at 1 hr and remained until the BrdU pulse at 15 hrs (white column on right). The 15 hr untreated control (gray column on right) indicates the maximum number of BrdU-labeled cells obtained. The means of triplicate counts of ∼200 cells/field+/−1 s. d. are shown. *C*) Asynchronous, logarithmically growing human MCF7 cells were treated with 50 µM DRB from time zero (squares), or not treated (circles), during a 24 hr period. BrdU was added at the beginning of the experiment and remained throughout the 24 hr period. At the indicated times, samples were fixed and processed for BrdU incorporation to determine the percentage of cells that had entered S-phase. The first time point was exposed to BrdU for 30 min before fixation. The means of triplicate counts of ∼250 cells/field+/−1 s. d. are shown.

We have previously reported that Balb/mouse keratinocytes (Balb/MK, or MK), like murine fibroblasts, also lose the requirement for *de novo* mRNA synthesis in late-G_1_ [30]. We used synchronized MK cells to re-examine the timing of when this transition to mRNA transcription independence occurs. MK cells are EGF dependent in their growth requirements and can be effectively synchronized and released into G_1_ using an EGF deprivation protocol [30]. Such EGF-synchronized MK cells moving through G_1_ into S-phase were exposed to DRB at several time points and allowed to progress (if they could) to the normal peak of S-phase (15 hrs post-release for MK cells), at which time they were pulsed with BrdU to determine the percentage of cells that were capable of entering S-phase in the presence of the DRB added at earlier times (diagrammed in Figure 1A). Parallel control cultures were pulsed with BrdU at the same time points to determine the percentage of MK cells in S-phase at each time point. In this manner, comparison of the BrdU index for DRB-treated cells at each time point to the BrdU index for control cells at each time point allows one to determine when in late-G_1_, relative to the G_1_-S transition, the population loses sensitivity to DRB. One benefit of designing the experiment this way is that it takes into account that the population of cells moves through G_1_ into S-phase in a quasi-synchronous Poisson distribution [30].

As shown in Figure 1B (right side), exposure of MK cells to DRB at 1 hr (early G_1_) effectively blocked progression into S-phase, confirming our previous results [30] that the DRB dose chosen was biologically potent (also see below) and that MK cells absolutely require mRNA synthesis in early-G_1_. Thus, *de novo* mRNA synthesis is rate-limiting for cell cycle progression in early-G_1_. Exposure to the carrier, DMSO, from 1 hr onward did not block progression of MK cells into S-phase (Fig. 1B, right side). The G_1_-S transition in EGF-synchronized MK cells occurs at 12 hrs in the population (defined when ∼50% of control cells are BrdU-positive), and the peak of DNA synthesis occurs at 15 hrs (Figure 1B, left side). In contrast to the inhibitory effect of early-G_1_ treatment with DRB, treatment with DRB at 9, 10, or 12 hrs had little or no effect on the ability of MK cells to enter S-phase, indicating that the cells transitioned to an mRNA synthesis independent state in late-G_1_ (Figure 1B, right side). Treatment with DRB at 8 hrs blocked ∼50% of the MK population from entering S-phase, which indicates that the transition to DRB insensitivity occurred ∼4 hrs prior to the transition of the MK population into S-phase (*i.e.*, G_1_-S). We conclude that MK cells require *de novo* mRNA synthesis in early G_1_, but transition to an mRNA synthesis independent state ∼4 hrs prior to the G_1_-S transition, consistent with our previous findings [30], [37].

### Human cells are also insensitive to suppression of mRNA synthesis 3–4 hours prior to the G_1_-S transition

Thus far, the lack of need for new mRNA synthesis in late-G_1_ has only been shown for murine cell types [28], [29], [30]. We next determined if the same were true of human cells, but also wanted to verify that such a phenomenon was not an artifact of synchronization *per se*. To address these questions, we utilized an effective and straightforward extrapolation approach described by Campisi and Pardee [28] in which asynchronous, logarithmically-dividing cells are analyzed for DRB sensitivity. In a log population, cells are present at all cell cycle stages, including some within a few hours of S-phase and others in early G_1_. Cells in a log population that are within a few hours of S-phase when exposed to DRB, but no longer sensitive to DRB, will continue to progress through late-G_1_ into S-phase and incorporate BrdU at similar rates to untreated control cells [28]. The time after DRB treatment when the rates of nuclear labeling begin to plateau and diverge from untreated control populations can be used to extrapolate back to the time in G_1_ when the population loses sensitivity to DRB [28].

The results in Figure 1C show that log MCF7 human breast cancer cells displayed an expected ∼28% BrdU labeling index at the start of the experiment (time 0). At this time, all plates were treated with BrdU, which was allowed to accumulate into nuclei as they entered S-phase during the course of the experiment. Half of the plates were untreated (controls; circles in Figure 1C), to show the rate of BrdU labeling index increase over time, while the other half of the plates were exposed to DRB. Figure 1C shows that, as expected, the percentage of nuclear labeling in control populations steadily increased during the course of the experiment, achieving almost 100% labeling by 24 hrs, after all of the cells had a chance to transit one cell cycle (and thus enter S-phase). In contrast, DRB-treated populations steadily increased alongside control cells for only the first 3–4 hours, after which time nuclear labeling plateaued and diverged from that of control cells (Figure 1C, squares). These results indicate that MCF7 cells in the log population that were at cell cycle positions more than 3–4 hrs prior to S-phase were sensitive to DRB and failed to progress into S-phase, while those that were within 3–4 hrs of the G_1_-S transition at the time of drug treatment were not sensitive to DRB and entered S-phase unabated. We conclude that human cells, like murine cells, lose the requirement for ongoing *de novo* mRNA synthesis 3–4 hrs prior to S-phase entry, and that this situation is independent of synchronization. Further, since transformed murine cells [28], tumor-derived human cells (shown here), and non-transformed mouse cells [28], [29], [30] all display this characteristic, the loss of requirement for *de novo* mRNA synthesis in late-G_1_ is independent of the species or transformation status of the cell.

### MCM loading occurs in the last 4 hrs of G_1_-phase in MK cells

Evidence in the literature has suggested that mammalian MCM proteins load onto preRCs during the latter part of G_1_-phase in cells released from quiescence [13], [14], [15], [16]. We wanted to determine for MK cells when MCM loading occurred, relative to the timing of sensitivity to DRB and the underlying need for mRNA synthesis, since knowledge of this relationship would have important implications for understanding the mechanisms controlling MCM loading and late-G_1_ progression into S-phase.

**Figure 2:**
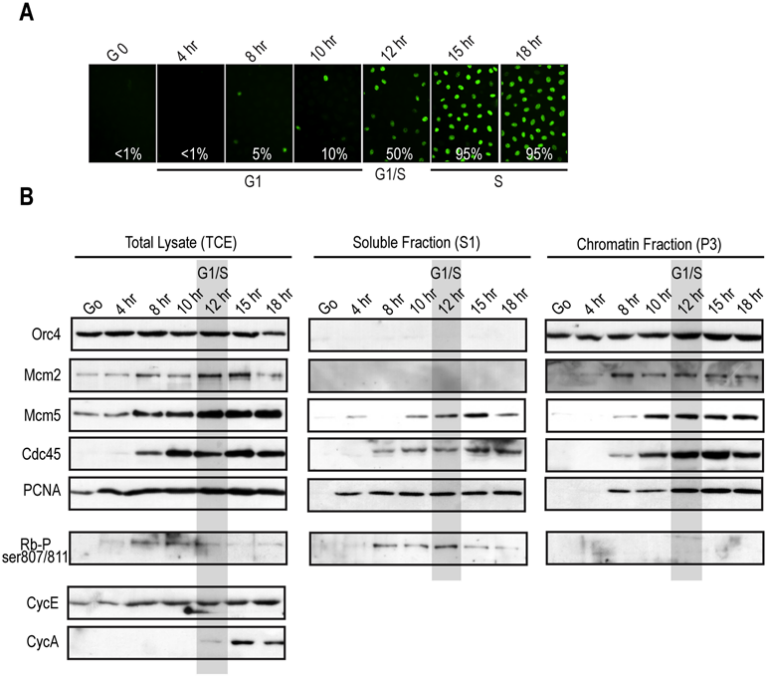
MCM, Cdc45, and PCNA load in the final 4 hrs of G_1_ in Balb/MK cells. (*A*) BrdU was pulsed into MK cells at the indicated times following release from quiescence to determine the kinetics of synchronization and entry into S-phase. (*B*) In parallel with the BrdU-pulsed samples in A, MK cells were collected at the indicated times and separated into total cell lysates (TCE), or fractionated into nucleosolic/cytosolic detergent-soluble extracts (S1) or chromatin-bound detergent-resistant extracts (P3). Immunoblotting with the indicated antibodies was performed on lysates from equal cell numbers loaded into each lane. The G_1_-S transition in MK cells (12 hrs after release) is overlayed in gray.

Assembly of preRCs onto chromatin templates (*i.e.*, at future origins of DNA replication) is operationally defined as the time when preRC subunits, particularly MCM subunits, display an increased presence on chromatin pellets based on their resistance to extraction with non-ionic detergents [11], [12], [14], [15]. To analyze the chromatin binding characteristics of preRC proteins, EGF-synchronized MK cells were released into G_1_ and allowed to progress into S-phase. At the times indicated, we collected total protein lysates (TCE), or fractionated separate samples into detergent-resistant (P3, chromatin) and detergent-sensitive (soluble/S1, cytosolic/nucleosolic) extracts [12], [14], [15]. Immunoblotting was performed to determine protein binding kinetics within each fraction/lysate over time. To verify effective fractionation, we immunoblotted against Lamin A/C, which partitions only with the chromatin fraction (data not shown, but see ref [14]). Parallel cultures of MK cells were pulsed with BrdU at the same time points to determine the kinetics of movement through G_1_ into S-phase (Figure 2A). The G_1_-S transition, as in the above experiment (Figure 1B), occurred at 12 hrs post-release.

Analysis of the dynamics of Orc4 revealed that it was present throughout G_1_- and S-phases at relatively steady levels (Figure 2B, TCE), and it was completely chromatin-bound at all times (Figure 2B, chromatin fraction), as seen for ORC in other studies [11], [12], [14]. MCM loading, as measured by the analysis of Mcm2 and Mcm5 dynamics, became visible starting at 8 hrs (Figure 2B, chromatin fraction). While Mcm2 appeared to load onto, and remain steadily bound to, chromatin from 8 hrs onward, Mcm5 chromatin binding clearly increased from the time period encompassing 8–12 hrs, after which it remained steady like Mcm2. Intriguingly, the MCM activators, Cdc45 and PCNA, both begin binding to chromatin at 8 hrs, with increasing chromatin association kinetics until 12 hrs (the G_1_-S transition), after which both were steadily chromatin bound throughout S-phase (Figure 2B, chromatin fraction). These results demonstrate that in MK cells released from quiescence, MCM, Cdc45, and PCNA loading onto chromatin occurs from 8–12 hrs, directly overlapping the time in late-G_1_ when *de novo* mRNA synthesis is no longer required for progression into S-phase. This further indicates that the underlying mechanisms regulating preRC assembly in the last few hours of late-G_1_ do not depend on production of new mRNA and are thus post-transcriptional in nature.

### Rb hyperphosphorylation coincides with MCM loading and the transition to an mRNA synthesis independent state in MK cells

The hyperphosphorylation of Rb in late-G_1_ is a well-established event that is commonly thought to regulate progression into S-phase by releasing E2F complexes that then cause increased *de novo* mRNA transcription of genes required for DNA replication [26]. A corollary of this is that the increased presence of new mRNA transcripts would likely comprise part of the mechanism governing preRC assembly. Since we established that MCM, Cdc45, and PCNA loading occurred in late-G_1_, and that this directly overlapped the time in late-G_1_ when *de novo* mRNA synthesis is no longer required by MK cells for entry into S-phase, we wanted to ascertain the kinetics of Rb hyperphosphorylation relative to these findings.

As described above, Rb phosphorylation during G_1_ progression involves the concerted action of two kinase complexes, Cyclin D/Cdk4 and Cyclin E/Cdk2 [20], [24]. The Cyclin D/Cdk4 complexes phosphorylate Rb as cells enter G_1_ after growth factor stimulation, rendering Rb hypophosphorylated and capable of binding to E2F and blocking E2F-mediated transactivation of promoters [21]. In late-G_1_, the Cyclin E/Cdk2 complexes further phosphorylate Rb, producing the hyperphosphorylated form of Rb that is associated with transcriptional upregulation events [21], [24], [25]. Importantly, the hyperphosphorylation of Rb occurs on several sites in Rb's C-terminal region (and in other regions), and such hyperphosphorylated Rb has been shown to become easily extractable with non-ionic detergents in late-G_1_ [24], [38], [39].

To determine the timing of Rb hyperphosphorylation in MK cells, we used an antibody specific for Rb phosphorylated on serines 807 and 811, both of which are in Rb's C-terminal region [39]. As can be seen in Figure 2B, Rb-ser807/811P appeared in MK cells at 8 hrs (Figure 2B, TCE), and as predicted [24], was completely detergent-extractable (Figure 2B, soluble fraction, note none in the chromatin fraction). Rb-ser807/811P remained present through late-G_1_ into S-phase. These results demonstrate that hyperphosphorylation of Rb in MK cells appeared around 8 hrs and was maintained thereafter into S-phase. Importantly, this indicates that Rb hyperphosphorylation appears in the MK cells coincident with when MCM, Cdc45, and PCNA loading commences, and Rb is hyperphosphorylated during their entire loading period in late-G_1_.

We also analyzed the expression of Cyclins E and A, both of which may play a role in late-G_1_ phosphorylation of Rb [20], [24], [25], [38], [40], [41]. Whereas Cyclin A appeared from the G_1_-S transition onward, Cyclin E was present in early-G_1_ and slightly increased at 8 hrs, the time of Rb-ser807/811P appearance (Fig. 2B). Although it is difficult to confirm the identity of *in vivo* kinases for Rb, these results are consistent with published studies implicating Cyclin E/Cdk2 as the catalytic complex producing late-G_1_ phosphorylation of Rb [20], [24], [25], [40], [41]. In addition, besides the Rb effects, the timing of the increased Cyclin E expression correlates nicely with the beginning of MCM loading, and Cyclin E is also known to elicit at least part of its cell cycle control through facilitation of MCM loading via phosphorylation of Cdc6 [17].

Even more intriguing, the hyperphosphorylation of Rb also occurs when the MK population transitions to the period in late-G_1_ when *de novo* mRNA synthesis is no longer rate-limiting for progression into S-phase (*i.e.*, at ∼8 hrs). Significantly, such a result is in direct opposition to the commonly-accepted paradigm suggesting that *de novo* mRNA transcription of a variety of genes is induced and required for progression into S-phase *after* Rb is hyperphosphorylated in late-G_1_. This indicates that the role of Rb hyperphosphorylation may extend beyond simple transcriptional control mechanisms in late-G_1_. Further, our results make it interesting to speculate that the control of MCM loading, or at least its timing, might comprise one potential regulatory target of Rb hyperphosphorylation in late-G_1_ (and we discuss other published studies consistent with this novel idea below). Indeed, MCM, Cdc45, and PCNA loading, as we have shown above (and further demonstrate below), is itself a transcriptional-independent process in late-G_1_, and the timing of their loading directly correlates with the appearance and persistence of hyperphosphorylated Rb.

### CHO cells lose the requirement for *de novo* mRNA synthesis 3 hours prior to S-phase

We wanted to determine if the timing of events described above for MK cells were also true for other mammalian cell types. To do this, we utilized Chinese hamster ovary (CHO) cells, which can be effectively synchronized, but using a very different regimen. Depriving CHO of isoleucine synchronizes the cells in a quiescent state, and synchronized CHO that are released into the cell cycle transit G_1_-S 9 hrs later, and reach an S-phase peak from 12–15 hrs [14], [15].

**Figure 3:**
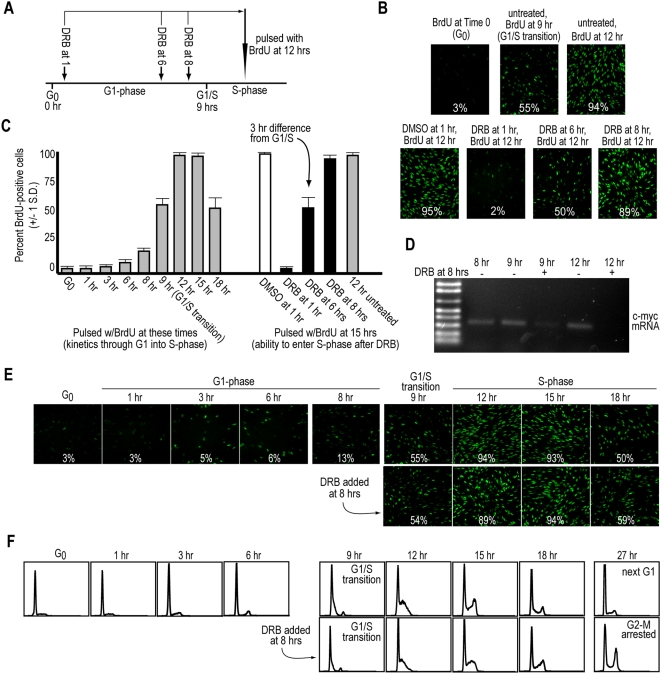
CHO cells do not require *de novo* mRNA synthesis during late G_1_, or for progression through S-phase. (*A*) Diagram illustrating the experimental design for the data obtained in *B&C*. (*B&C*) CHO cells were synchronized in G_0_ by isoleucine deprivation and then released into the cell cycle by re-addition of complete medium. At the times indicated, control cells were pulsed with BrdU to determine the kinetics of progression through G_1_ into S-phase (gray columns on left in *C*; examples shown in *B*). Cells treated with 50 µM DRB at the times indicated were allowed to progress to the peak of S-phase at 12 hrs, at which time they were pulsed with BrdU to determine the percentage of cells that could enter S-phase following different times of DRB exposure (black columns on right in *C*; examples shown in *B*). As a control, the DMSO carrier was added to a parallel culture at 1 hr and remained until the BrdU pulse at 12 hrs (white column on right in *C*). The 12 hr untreated control (gray column on right in *C*) indicates the maximum number of BrdU-labeled cells obtained without drug treatment. The means of triplicate counts of ∼200 cells/field+/−1 s. d. are shown. (*D*) RT-PCR analysis of c-*myc* mRNA levels on samples collected at the indicated times, with and without DRB exposure at 8 hrs. (*E*) Synchronized CHO cells were untreated (control, top row), or treated with 50 µM DRB at 8 hrs (bottom row), and pulsed with BrdU at each time point indicated in order to measure progression into and through S-phase. At least three fields of ∼200 cells were scored, and averages are displayed in panels with representative fields. Standard deviations (not shown) were within 1–5% for all panels. (*F*) Parallel to the samples in *E*, cells were collected and processed by flow cytometry using PI staining.

In order to determine when CHO cells lose sensitivity to DRB, synchronized CHO cells were treated with 50 µM DRB at 1, 6 and 8 hours following release into G_1_, and were allowed to progress (if they could) to the peak of S-phase (at 12 hrs) (diagrammed in Figure 3A). At this time, the plates were pulsed with BrdU to determine the percentage of cells that successfully progressed into S-phase after DRB treatment. Parallel control plates (no DRB added) were pulsed with BrdU at the hours indicated to determine the kinetics of progression through G_1_ into S-phase (Figure 3C, left side; examples are shown in Figure 3B).

Consistent with our results for MK cells, CHO cells in early-G_1_ were highly-sensitive to DRB, but not to the DMSO carrier, and failed to progress into S-phase when the drug was added 1 hr after release into G_1_ (Figure 3C, right side; examples are shown in Figure 3B). At 8 hrs after release, CHO cells were completely insensitive to the same dose of DRB and progressed into S-phase unperturbed with a BrdU-labeling index nearly identical to control cells (Figure 3B; and 3C, right side). At 6 hrs after release, treatment with DRB allowed only ∼50% of the population to enter S-phase (Figure 3B; and 3C, right side). Extrapolating back from the G_1_-S transition, which occurs at 9 hrs in the population (Figure 3C, left side), these results indicate that CHO cells lose sensitivity to DRB ∼3 hrs prior to entering S-phase (Figure 3C, right side). We conclude that CHO cells behave in a similar manner to that described above for MK cells (as well as for human [shown here] and other mouse cell types [28], [29], [30]), and lose the requirement for *de novo* mRNA synthesis in late-G_1_, in this case ∼3 hrs prior to entering S-phase.

### CHO cells enter S-phase on time, and progress through S-phase, when *de novo* mRNA synthesis is suppressed

We wanted to extend our observations thus far, which only demonstrate that *de novo* mRNA synthesis is no longer rate-limiting in late-G_1_ for progression of mammalian cells *into* S-phase. We asked whether suppression of ongoing mRNA production by DRB, beginning in late-G_1_, altered the timing of S-phase entry or the ability of cells to progress through and complete S-phase. CHO cells were synchronized and released into G_1_-phase. At 8 hrs (one hr prior to the G_1_-S transition) half of the cultures were treated with 50 µM DRB for the remainder of the experiment, and S-phase entry and progression were measured using BrdU incorporation and flow cytometry (Figure 3E&F). To verify that the DRB added at 8 hrs suppresses new mRNA synthesis in an acute manner (also see below), we analyzed the levels of c-*myc* mRNA using RT-PCR, since the c-*myc* transcript is known to be labile with a half-life of one hour or less [42], [43]. Accordingly, DRB treatment resulted in a noticeable suppression of c-*myc* mRNA within one hour, and a complete absence of the transcript by 12 hrs, when the cells were in S-phase (Figure 3D).

Analysis of BrdU labeling kinetics showed that control and DRB-treated populations were indistinguishable, and that both entered S-phase on time at 9 hrs (Figure 3E). Flow analysis confirmed this, showing the initial appearance of S-phase cells precisely at 9 hrs (Figure 3F). Relative to control cells, DRB-treated populations progress through S-phase unperturbed up to approximately 15 hrs (Figure 3E&F). We note that there is a continual presence of a 2N peak in every flow cytometric time point regardless of condition (Figure 3F). This is often visible for our CHO cells analyzed by flow cytometry and is likely due to a portion of the plated culture that does not release from the synchronization (but is collected for analysis), likely due to overly-dense regions on the periphery of the plate. In contrast, BrdU-analyzed fields are consistently gathered from central positions on the plate, where the cells display similar monolayer densities, and comparable synchronization and release dynamics.

From 15 hrs onward, the flow dynamics showed that DRB-treated cells began slowing somewhat relative to control cells, indicating they exited S-phase with delayed kinetics (Figure 3F). This is also evident in the BrdU-analyzed population, where more DRB-treated cells were still in S-phase at 18 hrs, relative to control cells (Figure 3E). However, as seen in a 27 hr sampling, when control cells have completed S-phase and have entered a new cycle, a significant portion of the DRB-treated cells did manage to finally exit S-phase, but accumulated with a 4N DNA content indicative of a G2-M arrest (Figure 3F). The latter is likely due to a need for synthesis of new mRNA species necessary for mitotic progression, such as that of Cyclin B [44]. We conclude from these results that, under conditions of mRNA synthesis suppression beginning in late-G_1_, CHO cells enter S-phase on time and progress through a significant portion of S-phase with normal kinetics (more than half of S-phase), and eventually exit S-phase, albeit with delayed kinetics.

### MCM loading in CHO cells occurs in late-G_1_ and overlaps the time when cells no longer require ongoing mRNA synthesis

**Figure 4:**
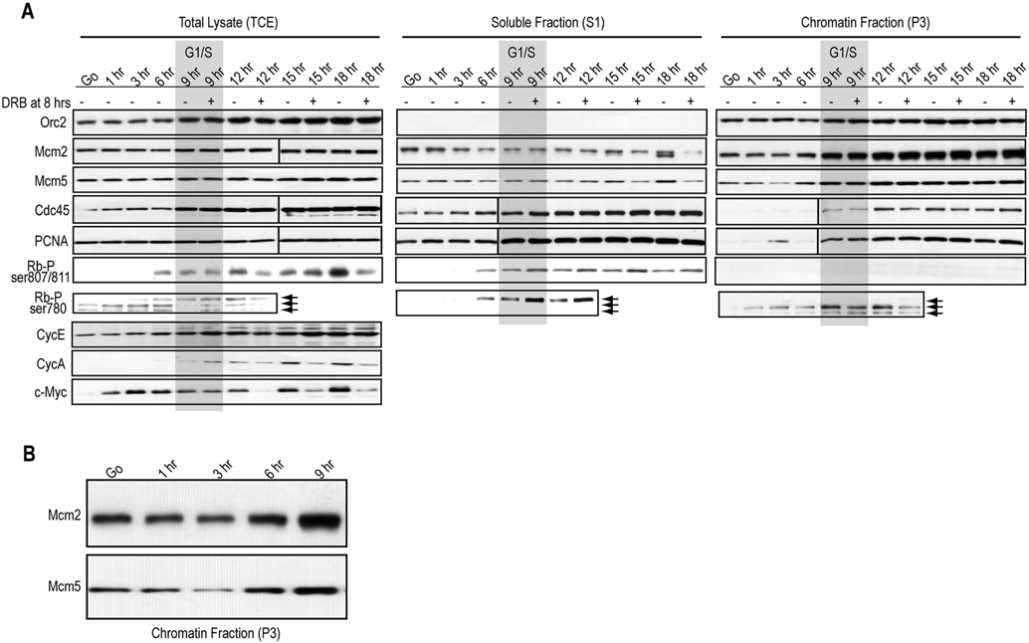
MCM, Cdc45, and PCNA load in the final 3 hrs of G_1_ in CHO cells. (*A*) Parallel to the BrdU and flow cytometry collection in Figure 3 E&F, CHO cells (half treated with DRB at 8 hrs) were collected and separated into total cell lysates (TCE), or fractionated into nucleosolic/cytosolic detergent-soluble extracts (S1) or chromatin-bound detergent-resistant extracts (P3). Immunoblotting with the indicated antibodies was performed on lysates from equal cell numbers loaded into each lane. The G_1_-S transition in CHO cells (9 hrs after release) is overlayed in gray. (*B*) An enlargement of the time points from part *A* for hours G_0_ through 9 is shown for Mcm2 and Mcm5 immunoblots.

We next assessed the dynamics of preRC subunits, particularly the MCM complex, on chromatin throughout G_1_ and S-phase in CHO cells relative to the window of time when *de novo* mRNA synthesis was required. Parallel to the BrdU and flow analyses in Figure 3E&F, we collected total protein lysates (TCE) and detergent-resistant (P3, chromatin) or detergent-sensitive (soluble/S1, cytosolic/nucleosolic) fractions [12], [14], [15]. Immunoblotting was performed on these fractions/lysates with the indicated antibodies (Figure 4).

The Orc2 protein was completely chromatin-bound in a relatively steady manner throughout the analysis (Figure 4A, chromatin fraction), similar to that shown above for Orc4 in MK cells. However, as for MK cells, the MCM complex displayed different binding kinetics as CHO progressed into late-G_1_ and S-phase. Mcm2 and Mcm5 were present at low steady levels in early-G_1_ (Figure 4A, G_0_ through 3 hrs), but were present on chromatin at noticeably higher levels during S-phase (Figure 4A, 9 hrs onward). Relative to the lower steady levels on chromatin at 3 hrs and before, Mcm2 and Mcm5 noticeably increased on chromatin at 6 hrs, and this increase continued until 9 hrs (*i.e.*, the G_1_-S transition) after which time it plateaued (Figure 4A&B). An enlargement of the MCM immunoblotting results for the chromatin fraction during the G_1_ period is shown in Figure 4B to help illustrate this transition. Cdc45 and PCNA also displayed differential chromatin binding characteristics as CHO progress into S-phase, but unlike that for MK cells, both proteins became chromatin-bound at the G_1_-S transition rather than in late-G_1_ (Figure 4A, chromatin fraction).

Suppression of *de novo* mRNA synthesis with DRB treatment one hour prior to the G_1_-S transition did not change the expression levels, nor chromatin-binding dynamics, of Orc2, Mcm2, Mcm5, Cdc45, or PCNA (Figure 4A). This is consistent with the fact that the CHO cells treated with DRB enter and progress through S-phase (Figure 3E&F). The expression of labile c-Myc protein, as a consequence of reduction of its coding mRNA (Figure 3D), was significantly reduced by the DRB treatment during the S-phase time points (Figure 4A, TCE, 12–18 hrs). The latter indicates that the DRB is highly effective and enduring in the experiment. We conclude from these results that MCM, Cdc45, and PCNA *maintenance* on chromatin during S-phase is not dependent on the ability of CHO cells to continually transcribe new mRNA. Further, since we established above that, beginning ∼6 hrs following release into G_1_, CHO cells no longer require *de novo* mRNA synthesis for progression into S-phase (Figure 3B&C), MCM, Cdc45, and PCNA *loading* onto chromatin during the final 6–9 hrs of G_1_ is also not dependent on *de novo* mRNA synthesis. Thus, MCM, Cdc45, and PCNA loading in late-G_1_ or at the G_1_-S transition is regulated at the post-transcriptional level in CHO cells, consistent with our findings in MK cells.

### Rb hyperphosphorylation coincides with MCM loading and the transition to an mRNA synthesis independent state in CHO cells

We wanted to determine the kinetics of Rb hyperphosphorylation in CHO cells as it related to the dynamics of MCM loading and the transition to an mRNA synthesis independent state in CHO cells. To do this, we analyzed the kinetics of appearance of two phosphorylated forms of Rb: Rb-ser807/811P and Rb-ser780P, using phospho-specific antibodies that could both recognize the CHO species of Rb.

As cells enter the cell cycle, the Cyclin D/Cdk4 complex is activated by growth factors and phosphorylates Rb in early-G_1_, producing an active hypophosphorylated form of Rb [21], [24]. One site on Rb that has been shown to be a substrate for Cyclin D/Cdk4 is serine 780 [41]. Consistent with these findings, Rb was indeed phosphorylated on serine 780 in early-G_1_ in CHO cells, and there were two prominent phosphorylated forms of Rb that contained this ser780P residue (Fig. 4A, TCE, G_0_-6 hrs, lower two arrows). At 6 hrs, a slower form of Rb-ser780P became more prominent, and this slower form was present throughout late-G_1_ and into S-phase (Fig. 4A, TCE, 9–12 hrs, uppermost arrow), in agreement with studies shown previously in CHO cells [45]. The slower form of Rb-ser780P is consistent with it being a hyperphosphorylated form of Rb due to its late-G_1_ appearance and slower mobility. Furthermore, hyperphosphorylated Rb is detergent-extractable [24], and the slower Rb-ser780P form of Rb is completely detergent-extractable (Fig. 4A, compare soluble fraction vs. chromatin fraction).

As a further confirmation that hyperphosphorylated Rb was present at 6 hrs, the C-terminally phosphorylated form of Rb, Rb-ser807/811P, was analyzed. Rb-ser807/811P clearly appeared at 6 hrs in CHO cells and remained present throughout late-G_1_, the G_1_-S transition, and S-phase (Figure 4A). As expected for hyperphosphorylated Rb, Rb-ser807/811P was completely detergent-extractable (Figure 4A, soluble fraction). We also analyzed the expression of Cyclins E and A, both of which may play a role in Rb phosphorylation in late-G_1_. Cyclin A appeared at the G_1_-S transition, several hours after the appearance of hyperphosphorylated Rb (Figure 4A). However, Cyclin E was present in early-G_1_ and slightly increased in expression at 6 hrs, the time when hyperphosphorylated Rb became prominent (Figure 4A). These results are consistent with evidence suggesting that Cyclin E/Cdk2 is involved in the late-G_1_ appearance of hyperphosphorylated Rb [20], [24]. We conclude from these results that Rb is hyperphosphorylated in CHO cells at ∼6 hrs after release into G_1_, coinciding with two important events: the increased loading of the MCM complex onto chromatin and the transition to an mRNA synthesis independent state in the cells. These findings in CHO cells are very consistent with that determined above for MK cells, where evidence of Rb hyperphosphorylation, MCM loading, and the transition to mRNA independence also overlap in the final third of G_1_.

Interestingly, except for perhaps a small increase in the presence of Rb-ser780P from 9 hrs onward (Figure 4A, only visible in soluble fraction), treatment with DRB one hour prior to S-phase had no effect on the dynamics of any of these phosphorylated forms of Rb, nor on Cyclin E expression (Figure 4A). However, although the appearance of Cyclin A at G_1_-S was not affected by DRB treatment, Cyclin A expression was reduced somewhat at later time points in S-phase by DRB (Figure 4A), perhaps contributing to the delay of exit from S-phase following DRB treatment of CHO cells (Figure 3E&F). Nonetheless, since CHO cells lose the requirement for *de novo* mRNA synthesis several hours prior to S-phase, we conclude from these results that the appearance, maintenance, and dynamics of hyperphosphorylated Rb and Cyclin E, as well as the initial appearance of Cyclin A, are regulated primarily at the post-transcriptional level in late-G_1_ and thereafter.

### Entry of CHO cells into S-phase is not dependent on induction of E2F-regulated mRNAs in late-G_1_

The coincident late-G_1_ timing of Rb hyperphosphorylation, MCM loading, and the transition to an mRNA synthesis independent state suggested, paradoxically, that for mammalian cells to assemble preRCs and successfully proceed into S-phase from this transition point, induction of new mRNA synthesis by E2F following ‘release’ from the Rb protein was unlikely to be required. To address this important question, we first verified that our DRB treatment was suppressing significant levels of mRNA synthesis as shown by prior reports, and then determined if several archetypal E2F-regulated mRNA targets are indeed suppressed by DRB in our experiments.

**Figure 5:**
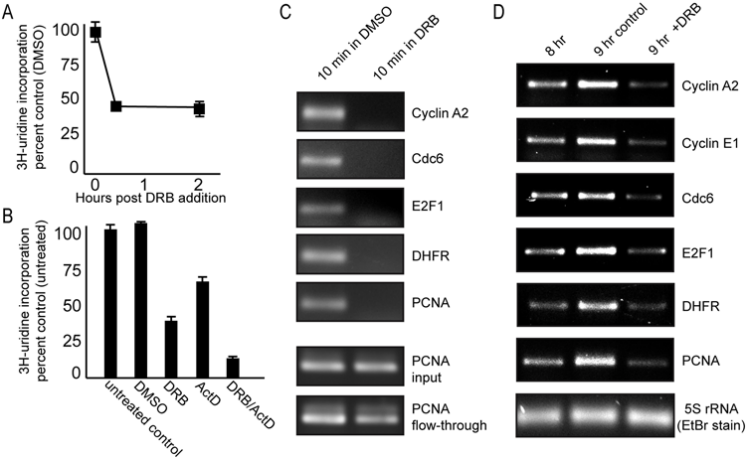
DRB effectively suppresses mRNA synthesis and acutely blocks the expression of E2F-reglated targets. (*A*) Asynchronous CHO cells were treated with 50 µM DRB or DMSO carrier at time 0, and [3H]-uridine (3 µCi/ml) was added to the cultures for 1 hr starting at 30 min or 2 hrs post treatment. For time 0 uridine pulse, no DRB or DMSO was added. TCA-precipitable counts were determined by a scintillation machine for duplicate samples, and the averages were plotted+/−1 s.d. as percentages of the DMSO control. (*B*) CHO cells were treated with DRB (50 µM), DMSO, ActD (0.05 µg/ml), or DRB+ActD for 30 min, and were then labeled with [3H]-uridine for 1 hr. TCA-precipitable counts were determined as above. (*C*) *In vivo* RNA run-off assay. DRB was exposed to CHO cells for 10 min, then total RNA was labeled for 1 hr with BrU (100 µM). BrU-labeled RNA was purified as described in the Methods, and then analyzed by RT-PCR using primers against the E2F targets indicated. Input and flow-through samples were analyzed by RT-PCR for PCNA presence to verify loading and confirm that RNA was not degraded before, during, or after the IP step. (*D*) RNA samples from the experiment in Fig. 3D were subjected to RT-PCR analysis using primers against the E2F targets indicated. 5S RNA is shown as a loading control, as it is much less susceptible to DRB suppression than mRNA.

DRB is a potent and selective inhibitor of RNA polymerase II and has been shown at doses similar to ours to acutely inhibit production of 95% of cellular mRNA (polyadenylated RNA) by 3–5 minutes of exposure to mammalian cells [35]. Used in this manner, DRB has little effect on rRNA and tRNA production [35], both of which must be synthesized by RNA polymerases I and III, respectively, for cell viability [28], [30], [35]. Using [3H]uridine incorporation assays (into newly-synthesized RNA of all types), Darnell and colleagues showed clearly that treatment with DRB alone produces an ∼60% reduction in total uridine incorporation that is attributable to suppression of specifically mRNA synthesis. The remainder of uridine incorporation is attributable to rRNA and tRNA production, but mostly that of rRNA [35]. Using the same approach, we show in Figure 5A that exposure of CHO cells to 50 µM DRB results in a very similar reduction of total uridine incorporation (just over 50%) that is maximal at (or prior to) 30 minutes post treatment, the latter consistent with the acute nature of DRB-mediated suppression of RNA pol II activity.

Low doses of Actinomycin D (0.05 µg/ml) specifically block rRNA production without affecting mRNA production [35], [36], [46], [47]. To demonstrate that the remainder of uridine incorporation not suppressed by DRB is primarily attributable to rRNA synthesis, we performed another experiment based on that done by Darnell and colleagues [35]. CHO cells were treated with DRB (50 µM), ActD (0.05 µg/ml), or DRB+ActD for 30 minutes, and then uridine incorporation was analyzed (Fig. 5B). DRB alone produced an ∼63% reduction of uridine incorporation, while ActD alone suppressed ∼35% of the incorporation. DRB and ActD together produced an ∼90% reduction, demonstrating as before [35] that the two pools of uridine suppression are distinct and the effects of the drugs are additive. We conclude from these results that DRB indeed suppresses a significant amount of mRNA synthesis in CHO cells, with the remainder of RNA production not blocked by DRB due primarily to rRNA synthesis.

We next verified that several well-documented E2F target genes/mRNAs are indeed acutely suppressed by DRB in our CHO experiments. In the first approach, we treated CHO cells with DRB or DMSO for 10 min, then pulsed cells with bromo-uridine (BrU) to label newly-synthesized RNA (an *in vivo* RNA run-off assay). Total RNA was collected and nascent RNA was further purified using anti-BrdU antibodies (which cross-react with BrU), converted to cDNA, then subjected to PCR amplification using primers to several E2F targets. Figure 5C shows that after just 10 min of DRB exposure, the production of new Cyclin A2, Cdc6, E2F1, DHFR, and PCNA mRNAs is efficiently blocked by DRB (we also tried to test for Cyclin E1 expression in this assay, but could not detect any signals after purification; however, Cyclin E1 was examined in the next experiment). We analyzed input and flow-through samples for PCNA signal to verify that RNA degradation had not occurred during any part of the purification procedure (Fig. 5C, bottom). This indicates that, consistent with the Darnell report [35], RNA polymerase II activity and the promoters for these well-established E2F targets, in particular, are significantly suppressed almost immediately after DRB exposure in our experiments.

Finally, we verified that DRB does indeed acutely block the induction of new mRNA synthesis of these same E2F targets in our CHO synchronization experiment (Fig. 3), while at the same time having no negative effect on the ability of the cells to enter S-phase. We already showed that c-*myc* mRNA expression (c-*myc* is also E2F-regulated) is acutely blocked after treatment with DRB prior to S-phase entry (Fig. 3D). Using the same RNA samples for PCR, we find that the induction of Cyclin A2, Cyclin E1, Cdc6, DHFR, PCNA, and E2F1 coding mRNAs is also acutely blocked by DRB added in late-G_1_ (Fig. 5D), but the cells nonetheless successfully approach and pass through the G1-S transition (Fig. 3). In fact, the levels of each of their mRNAs may even be lower after DRB exposure (likely due to normal degradation of remaining molecules). Note that in control samples across 8–9 hrs these mRNAs are clearly seen to increase, as predicted, as cells approach and enter S-phase. We conclude from these results that CHO cells do not require *de novo* synthesis of mRNA in the last few hours of G_1_, after Rb hyperphosphorylation, and that, in particular, the subsequent induction of E2F-regulated genes at the mRNA level is not strictly required for progress into S-phase. One possible concern with this interpretation is that untested or unidentified E2F-regulated genes may exist that are insensitive to DRB but must be induced for S-phase entry. This possibility is very unlikely, however, as it has been shown that E2F transactivation in general is not required for cell cycle progression [48], a finding that is completely consistent with those presented here, and which is discussed in more detail below.

## Discussion

We have carefully defined the kinetics of several important cell cycle regulated, as well as cell cycle regulating, events that occur as mammalian cells progress through G_1_ into S-phase. Several conclusions can be drawn from these experiments, with significant implications for understanding how progression into S-phase is controlled in late-G_1_. Studies dating back almost three decades have suggested that new mRNA synthesis is not required by mammalian cells in the late-G_1_ period, while separate work from other studies has established a general paradigm in which Rb becomes phosphorylated in late-G_1_, freeing E2F to induce new transcription of mRNA for genes required for S-phase entry. Clearly, these concepts appear to be in opposition to one another, and one explanation for such conflicting information may derive from the fact that these concepts have been developed in separate studies using different cell lines and non-overlapping analyses. Importantly, some of the novelty of the experimental results we have shown here derives from the fact that, in contrast to prior studies, we obtained our results from comprehensive experiments in which multiple concepts were co-analyzed to determine if predictions from the literature were valid.

**Figure 6:**
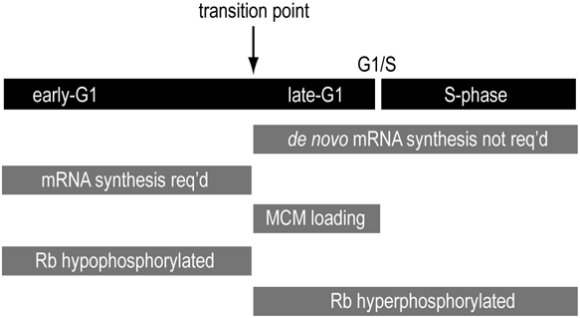
Summary of results described in this report. Mammalian cells require ongoing mRNA synthesis in the first part of G_1_-phase. Concurrent with this timeframe, the Rb protein is hypophosphorylated and MCMs have not loaded onto chromatin at the preRCs. In the final 3–4 hours of G_1_-phase, mammalian cells pass through a transition when Rb is hyperphosphorylated, MCMs load onto chromatin, and new mRNA synthesis is no longer rate-limiting for MCM, Cdc45, or PCNA loading, nor for the eventual progression of the cells into and through S-phase.

In MK cells, which have a 12 hr G_1_-phase, MCM loading occurs from 8–12 hrs post-release. In CHO cells, which have a 9 hr G_1_-phase, MCM loading occurs from 6–9 hrs post-release. Thus, in both cell types, MCM loading occurs in the final 33% of G_1_-phase (Figure 6), which is consistent with the predictions that can be made from the results of Mailand and Diffley indicating that Cyclin E/Cdk2 activity in late-G_1_ is required for phosphorylation/stabilization of Cdc6 to achieve MCM loading during such time [17]. While the concept that MCM loading occurs in this latter part of G_1_ can be predicted from the Diffley study [17] and from other studies [13], [14], [15], [16], when one considers the relationship of this information to the timing of Rb hyperphosphorylation and the transition to mRNA synthesis independence (both sets of information obtained in parallel, and discussed below), several significant conclusions can be drawn that have important implications for understanding how mammalian cells control late-G_1_ progression into S-phase.

We demonstrate that, as predicted from several independent studies, at the time MCM complexes load onto chromatin in late-G_1_, at least two important transitions do indeed occur that have regulatory implications for MCM loading. In both MK and CHO cells, MCM loading coincides with the appearance of hyperphosphorylated Rb and with the transition to an mRNA synthesis independent state in the cells (Figure 6). This indicates that the ongoing synthesis of new mRNA transcripts in late-G_1_ is not required to regulate MCM loading onto chromatin via control of other factors required for this process. Indeed, in both cell types analyzed here, MCM subunits are present even in early-G_1_ prior to this transition, and in CHO cells in particular, the total protein levels of MCM subunits do not fluctuate at all during G_1_ or S-phase in the presence of DRB (Figure 4, TCE samples). A similar situation exists for Cdc45 and PCNA, where their expression and loading are also independent of *de novo* mRNA synthesis in late-G_1_. One possible outcome of these experiments might have been that preRC assembly or dynamics *was* affected by the block to new mRNA synthesis, but that any reduction in preRC assembly/dynamics had no effect on entry into S-phase. Clearly, our results show that this is not the case, and that blocking new mRNA synthesis in late-G_1_, concurrent with the timing of preRC assembly, has no observable effects on any measured aspect of preRC dynamics. As a corollary, these data suggest that any mRNA transcripts required for MCM, Cdc45, or PCNA protein translation, or for other factors involved in the loading of these proteins onto chromatin, would likely have been made at sufficient levels *prior* to time when cells lose their requirement for new mRNA production and begin MCM loading.

Given the concepts just described, it is particularly intriguing that the appearance of hyperphosphorylated Rb coincides not only with the start of MCM loading, but also with the late-G_1_ transition to a cell cycle state that no longer depends on *de novo* mRNA synthesis for progression into S-phase. Independent reports in the literature predicted that these overlaps might exist, and we have now shown direct experimental evidence that these overlaps do indeed exist. It is a generally assumed paradigm that transcriptional activation mechanisms involving new mRNA synthesis control traverse through late-G_1_ following hyperphosphorylation of Rb and release of E2F complexes [26]. Indeed, there is a wealth of evidence in the literature demonstrating that transcription of numerous genes, whose protein products are important for G_1_-S transit, is controlled by Rb-E2F in a positive manner [25], [49], [50], [51], [52], [53], [54], [55], [56].

As a corollary to this paradigm, though, such E2F-regulated transcription (and mRNA synthesis in general) *should* be rate-limiting in late-G_1_ for progress into S-phase. To date, however, no studies have demonstrated that E2F-regulated transactivation in late-G_1_, while definitely occurring, is truly *required* to occur. Here, we have addressed this question for the first time. In direct opposition to this paradigm, we show that new mRNA synthesis is not rate-limiting for entry into S-phase, at a time that coincides with, and continually overlaps thereafter, the appearance of Rb hyperphosphorylation. Furthermore, we show direct evidence that multiple well-documented E2F targets can be acutely blocked by DRB from being transactivated in late-G_1_, but mammalian cells nonetheless continue into S-phase unhindered. Thus, although up-regulation of new mRNA after hyperphosphorylation of Rb and E2F ‘freeing’ in late-G_1_ clearly does occur, the cells are not dependent on *de novo* late-G_1_ production of these new mRNA transcripts for successful progression into S-phase. Furthermore, our data even demonstrates that mammalian cells can progress through much of S-phase unperturbed when *de novo* mRNA synthesis is continually suppressed from late-G_1_ onward.

Consistent with the work presented here, Dean and colleagues provided paradoxical evidence that E2F1-mediated transcriptional activation *per se* is not required for cell cycle progression [48]. Ectopic expression of an E2F1 protein that is lacking its transactivation domain, and predicted to act as a dominant-negative protein because it cannot induce transcription of important genes required for cell cycle progression, instead allows normal cell growth [48]. Rather than transcriptional activation being an important regulatory mechanism of E2F1, it was instead shown that transcriptional downregulation by E2F1, via Rb recruitment, was the important function of E2F1 (when cells were subjected to negative growth signals) [48]. Thus, although E2F1 is capable of upregulating transcription, transcriptional activation *per se* by E2F1 is paradoxically not required by cells at any time in the cell cycle [48]. Such results also argue strongly that even if we have missed certain E2F-regulated targets in our analysis (it is not practical to examine all E2F targets, as some may not yet have been identified), it is highly unlikely that transcriptional *upregulation* of any E2F-regulated genes in late-G_1_ is required for entry into S-phase.

Other intriguing evidence exists demonstrating that Rb suppression of entry into S-phase is separable from negative effects on transcriptional control by E2F1 [57]. In Saos-2 cells that are lacking Rb, expression of Rb causes a G_1_ arrest and an expected decrease in the mRNA levels of E2F1-regulated genes such as cyclin A, E, and E2F1, consistent with the Rb-E2F1 model of transcriptional downregulation [26], [57]. However, the expression of the protein products encoded by these genes is not acutely suppressed even though the cells fail to enter S-phase [57]. Thus, although E2F1-sensitive transcription is indeed suppressed by Rb expression, these results strongly argue that E2F1-mediated transcriptional control over the presence of these proteins does not acutely regulate G_1_-S progression. Importantly, both of the studies discussed above [48], [57] are in complete agreement with our data presented herein indicating that in late-G_1_, transactivation of new mRNA species by E2F1, or by other transcription factors, is not required for successful G_1_-S progression, including the final assembly and activation of preRCs.

Given the problems described above with the conventional paradigm that Rb-E2F transcriptional events control G1-S transit in late-G_1_, it appears that the role of Rb-E2F in late-G_1_ needs to be revisited. Intriguingly, the coincidence of Rb hyperphosphorylation and MCM chromatin loading makes it interesting to speculate that a regulatory relationship may exist between these two events. Hyperphosphorylation of Rb may constitute a trigger in late-G_1_ that facilitates MCM loading, in a manner that does not depend on new mRNA production to achieve MCM loading and G_1_-S transit. Indeed, blocking Rb hyperphosphorylation by overexpressing the Cdk4 inhibitor p16 results in a block toward MCM loading that is dependent on the presence of Rb [58]. Thus, MCMs cannot load when Rb is hypophosphorylated, but we show here that MCMs do load coincident with, and overlapping, Rb hyperphosphorylation. Furthermore, the Orr-Weaver lab has shown that metazoan Rb and E2F (in flies) control replication origin activity through interaction with the preRC/ORC [59], [60]. Significantly, this control over preRC/origin activity in these studies was via a direct interaction between Rb-E2F and the preRC components, and did not involve any *de novo* mRNA regulation by Rb-E2F [59], which is completely compatible with the results shown here. While validation of this potential regulatory relationship in mammalian cells will require further mechanistic investigation well beyond the scope of this study, the results presented here clearly demonstrate that mammalian cells transition into a unique preRC assembly state in late-G_1_ that is controlled by post-transcriptional mechanisms following Rb hyperphosphorylation.

Finally, aside from the concepts discussed above, there is another result from these studies that merits some analysis. In CHO cells, Cdc45 and PCNA loading occurs prominently at the G_1_-S transition. However, in MK cells, Cdc45 and PCNA loading overlap MCM loading during the final 4 hrs of G_1_, and thus occurs noticeably earlier than the G_1_-S transition. Cdc45 recruitment has been shown to be dependent on the activity of Cdk2 and Cdc7 kinases in metazoans [61], and in yeast, recruitment of Cdc45 along with specific phosphorylation events triggers G_1_-S transit at the molecular level [62], [63]. Since we demonstrate that mammalian Cdc45 and PCNA can be recruited to chromatin several hours prior to G_1_-S (at least in MK cells), it appears that the recruitment *per se* of Cdc45 and PCNA in somatic mammalian cells does not itself constitute a G_1_-S trigger. This suggests that further phosphorylation events, perhaps similar to those identified in yeast, but independent of phosphorylation that is required for Cdc45 and PCNA chromatin loading, are likely required to initiate S-phase in mammalian cells.
